# Pre-Ebola virus disease laboratory system and related challenges in Liberia

**DOI:** 10.4102/ajlm.v5i3.508

**Published:** 2016-10-31

**Authors:** Stephen B. Kennedy, John B. Dogba, Christine L. Wasunna, Philip Sahr, Candace B. Eastman, Fatorma K. Bolay, Gloria T. Mason, Mark W.S. Kieh

**Affiliations:** 1 Incident Management System, Emergency Operations Center, Ministry of Health, Monrovia, Liberia; 2 Partnership for Research on Ebola Virus in Liberia, Liberia-US Clinical Research Partnership Program, First Floor, John F. Kennedy Medical Center, Monrovia, Liberia; 3 National Reference Laboratory, Ministry of Health, Charlesville, Margibi County, Liberia; 4 Africabio Enterprises, Inc., Payne Avenue, Sinkor, Monrovia; 5 Liberia Institute for Biomedical Research, Ministry of Health, Charlesville, Margibi County, Liberia; 6 National Research Ethics Board, Partnership for Research on Ebola Virus in Liberia, First Floor, John F. Kennedy Medical Center, Monrovia, Liberia

## Abstract

Prior to the Ebola virus disease outbreak in Liberia, the laboratory system was duplicative, fragmented and minimally coordinated. The National Reference Laboratory was conceptualised to address the existing challenges by promoting the implementation of effective and sustainable laboratory services in Liberia. However, in a resource-limited environment such as Liberia, progress regarding the rebuilding of the health system can be relatively slow, while efforts to sustain the transient gains remain a key challenge for the Ministry of Health. In this paper, we describe the pre-Ebola virus disease laboratory system in Liberia and its prevailing efforts to address future emerging infectious diseases, as well as current Infectious diseases, all of which are exacerbated by poverty. We conclude that laboratory and diagnostic services in Liberia have encountered numerous challenges regarding its efforts to strengthen the healthcare delivery system. These challenges include limited trained human resource capacity, inadequate infrastructure, and a lack of coordination. As with most countries in sub-Saharan Africa, when comparing urban and rural settings, diagnostic and clinical services are generally skewed toward urban health facilities and private, faith-based health facilities. We recommend that structured policy be directed at these challenges for national institutions to develop guidelines to improve, strengthen and sustain diagnostic and curative laboratory services to effectively address current infectious diseases and prepare for future emerging and re-emerging infectious diseases.

## Introduction

The primary focus regarding the implementation of effective and sustainable laboratory services in Liberia was accentuated with the conceptualisation of the National Reference Laboratory (NRL) in 2008.^1^ Initially, the NRL was formulated, with an agenda for future expansion, as a single-room facility housed within the National Drug Service at the John F. Kennedy Memorial Hospital, a tertiary referral hospital in Monrovia. With prevailing viral infectious diseases as its initial core, its primary laboratory diagnostic capacity was limited to measles, rubella and yellow fever. At the time, samples for Lassa fever were collected, packaged and transported for diagnosis at the Lassa Fever Laboratory in Kenema, Sierra Leone. With support from international partners, basic equipment and human resource development aimed at expanding the capacity of the NRL were secured, and the laboratory was subsequently relocated in 2009 to the compound of the Liberia Institute for Biomedical Research in Charlesville, Lower Margibi County. Afterward, a programme known as the National Diagnostic Unit (NDU) was established.

Liberia has gradually been rebuilding its healthcare delivery system after 16 years of devastating civil conflicts which erupted in December 1989, destroyed the country’s health infrastructure^2^ and subsequently culminated with a drastic brain drain as the result of the migration of trained Liberians to politically-stable countries. In a resource-limited environment such as Liberia, progress regarding the rebuilding of the health system has been relatively slow and sustaining the gains from transient spikes in improved health indicators remain a major challenge.

Accordingly, laboratory-based service delivery in Liberia has been basically defined in several key policy documents^1,2,3^ of the Ministry of Health that established the minimum threshold for trained human resources development, laboratory infrastructure, disease surveillance and laboratory diagnostic capacities at each level of the healthcare delivery system in the country.

## Laboratory structures

Liberia’s laboratory system was initially classified into a three-tiered health system structure consisting of the: (1) National; (2) County (main political sub-divisions); and (3) Peripheral (health centres and clinics) levels.^1,2,3^ The National level comprises the NRL and NDU; the County level involves the referral hospitals in the political subdivisions of the country; and the Peripheral level accounts for the district and community health facilities. With the development of the Country’s National Decentralization Policy^2,4^ for the regionalisation of services within the country, geared toward the improvement of services in the disenfranchised rural areas, a fourth level of the laboratory system was incorporated; thereby, allowing for (1) National; (2) Regional; (3) County; and (4) Peripheral levels. Accordingly, the NRL and NDU are strategically situated within the National level. The regional tier consists of five regional laboratories based on the decentralisation of the country, namely: (1) Southeast A; (2) Southeast B; (3) North central; (4) South central; and (5) Western. The county and peripheral tiered systems are basically the same levels as in the prior three-tiered approach.

## Laboratory services

In general, there are two main categories of laboratory services in Liberia. The first is the clinical laboratory services designated for clinical specimen testing for diagnostic purposes and the second is public health laboratory services directed at disease surveillance.

According to its mandate, the NRL has a four-fold responsibility: (1) perform diagnostic testing services on specimens for diseases with epidemic potential that are under surveillance, especially those that are immediately notifiable to the World Health Organization, now inclusive of Ebola virus disease (EVD), as required by the International Health Regulations; (2) oversee the network of public laboratory structures within the country and the provision of services through quality assurance, training, monitoring and supportive supervision; (3) assess new and available technologies; and (4) provide technical advice to the Ministry of Health.

Similar to the NRL, the NDU conducts quality assurance, monitoring and evaluation, and training. Furthermore, it coordinates procurement processes for the distribution of laboratory commodities for special programmes supported by the Global Funds, such as HIV, AIDS, malaria and tuberculosis programmes. When constructed, the regional laboratories will support the NRL in its surveillance testing (i.e., its mandate for public health laboratory services), as well as the county-level coordination of the activities of the laboratory system. In addition, they will lend diagnostic support to the laboratories at the county hospitals.

These structures are responsible solely for clinical specimen testing for diagnostic purposes. Test categories include, among others, chemistry, haematology and parasitology. Apart from the traditional structures indicated for service delivery, certain programs of the Ministry of Health are also involved in cross-cutting testing activities (laboratory clinical diagnosis and public health surveillance purposes). These programmes include: (1) the National AIDS Control Program; (2) the National Malaria Control Program; (3) the National Tuberculosis & Leprosy Control Program; and (4) the National Blood Safety Program.

Laboratory equipment, reagents and supplies for the operations of the various laboratory structures are either procured by the Ministry of Health or donated by international partners. The operation of the procurement activities is performed by two organisations: (1) the National Drug Service; and (2) the Global Fund, which supports the HIV, tuberculosis and malaria programmes. Both of these key operational processes (procurement and donations) for the effective functionality of the laboratory system in Liberia exist without defined ‘Maintenance and/or Service Contracts’.

## Laboratory workforce development

There are two accredited faith-based and one publicly-owned laboratory training institutions in Liberia. These are: (1) the Mother Patern College of Health Sciences in Monrovia, Montserrado County; (2) the Phebe School of Nursing in Suacoco, Bong County; and (3) the Tubman National Institute of Medical Arts in Monrovia. The qualifications offered by these training institutions, based on a duration of training that ranges from 2–4 years, includes Diploma in Laboratory Science (Tubman National Institute of Medical Arts and Phebe School of Nursing); Associate of Science and Bachelor of Science in Laboratory Technology by the Mother Patern College of Health Sciences.

## Laboratory system

The status of the existing laboratory structure and service delivery system in Liberia is characterised by numerous challenges, which were uncovered during the EVD outbreak in Liberia that became a major public health problem in the country and the sub-region.^5,6^ Poor health infrastructure, lack of logistics during the beginning of the outbreak, and an inadequate number of trained staff in public health disease surveillance and laboratory diagnostic response in emergency situations, were key factors leading to the delay in response to the outbreak. These pre-EVD laboratory challenges, among others, included:

- Lack of integration between public health surveillance and laboratory diagnostic services, resulting in fragmented service delivery.
- Lack of a well-defined organisational structure with clearly-defined objectives, including existing duplications of roles and responsibilities within the tiered laboratory systems to support an effective leadership and management system.
- Lack of adequate financial resources for infrastructure support and operational costs.
- Inadequately trained laboratory workforce, including inadequate deployment across the country.
- Unavailability of maintenance and service contracts for procured and/or donated laboratory equipment and supplies to sustain the effective operation of the laboratory infrastructure.
- Lack of adequate biomedical equipment technicians to maintain and repair laboratory equipment.
- Unstable electric power supply and inadequate water supply for laboratory service utilisation.
- Lack of effective Laboratory Information Management System for coordination, operations, and the efficient functionality of the various tiered systems.

### Lack of a well-organised quality management scheme

Despite these challenges, efforts were directed at the development of a National Laboratory Policy to prioritise the delivery of laboratory services within the health system.^7^ It was aimed at addressing the challenges associated with limited diagnostic services, which subject patients to inappropriate treatment, chronic illnesses, high out-of-pocket expenditures on healthcare, loss of income and, ultimately, loss of confidence in health services. To mitigate the challenges, the proposed policies targeted the following areas:

1. **Organisation:** Strengthen the legal and regulatory framework, as well as the technical and administrative organisational structures, for the delivery of laboratory services nationwide.
2. **Test selection and referral linkage:** Ensure that appropriate tests are performed at the appropriate facility level in a decentralised network and the provision of oversight to establish appropriate communication structure and processes in order to create a referral linkage or network between facilities.
3. **Human resources:** Ensure the presence of certified laboratory personnel in all public and private laboratories, regulate through periodic certification and develop curriculum standards to support training institutions.
4. **Quality assurance:** Ensure that laboratories are certified according to defined standards with established systems for independent certification.
5. **Infrastructure:** Provide safe and adequate infrastructure standards for all public and private laboratories at each level of care and ensure, through certification, that laboratories have standard operating procedures and required infrastructure for laboratory safety and operation.
6. **Equipment:** Ensure that all laboratories have properly working equipment with both preventative and curative maintenance, and enforce standardisation such that all equipment and supplies meet national specifications and are maintained according to manufacture guidance.
7. **Supply chain management:** Support an uninterrupted distribution of reagents and supplies to all laboratories, and ensure that an effective system is in place to select, quantify, procure, transport, store, distribute and keep records of all equipment, reagents, and supplies.
8. **National blood safety:** Ensure a safe supply of blood for transfusions and promote voluntary blood donations.
9. **Biosafety:** Provide a safe environment and ensure laboratory personnel adhere to safety protocols and quality assurance standards, including laboratory safety for protection of professional staff, public and the environment.
10. **Financial resources:** Allocate resources to sustain quality laboratory services.
11. **Research and development:** Identify major laboratory research priorities and promote the culture for operational research, and evaluate and introduce new innovative technologies into the laboratory system.
12. **Collaboration:** Improve laboratory services through public and private partnerships, as well as support for national and international collaborations.
13. **Ethics:** Ensure a professional code of conduct and client confidentiality.
14. **Information system:** Design a laboratory information system that is fully integrated for information flow between laboratories within a referral network.
15. **Policy implementation:** Monitor and evaluate the efficiency and effectiveness of the laboratory system, as well as its sustainability.
16. **Policy regulation:** Ensure that laboratory guidelines are updated regularly and in compliance with internationally-accepted standards.

### Recommendations

We recommend that a joint public–private partnership team be established to conduct a comprehensive technical assessment of the laboratory system within the country and provide appropriate recommendations to combat emerging infectious diseases. Enhancing the laboratories’ capacity for research requires: the availability of essential equipment and appliances; development of relevant and sustained human capacity through continuing education, on-the-job training and mentorship; formulation and adherence to guidelines and standard operating procedures, and compliance with Good (Clinical) Laboratory Practice and other international standards, such as ISO 15189.

**FIGURE 1:**
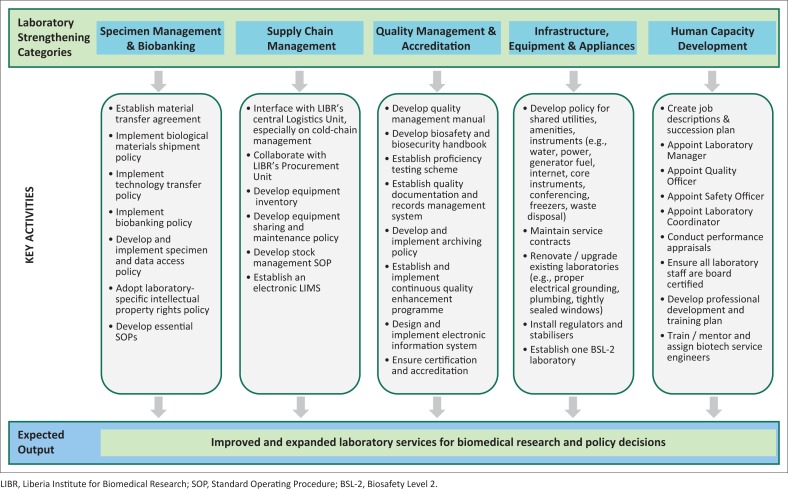
Proposed mechanism for strengthening the laboratory system in Liberia.

The EVD outbreak in Liberia has necessitated improved laboratory capacity to respond to an epidemic of emerging infectious pathogens as well as support for the development of a suitable research platform during such public health emergencies. Accordingly, we propose a framework to strengthen the laboratory system of the country based on a preparedness strategy to mitigate future outbreaks of emerging and re-emerging infectious diseases (See Figure 1).

### Conclusion

Laboratory and diagnostic services in Liberia have encountered numerous challenges regarding their contributions toward the strengthening of the healthcare delivery system in Liberia. These challenges are a consequence of limited trained human resource capacity, inadequate infrastructure, and lack of coordination among the four tiers. Diagnostic services are skewed in urban health facilities as compared to those in rural communities, and also skewed in favour of private and faith-based health facilities when compared to public health facilities. These disparities are markedly observed throughout the country and significantly impact patient care and emergency situations. The lack of in-country diagnostic laboratories for the diagnosis of viral haemorrhagic fevers, such as Lassa fever and EVD, prior to the EVD outbreak, confirmed the limitations and challenges the country faced with regard to diagnostics services. Prior to the EVD outbreak, suspected Lassa fever specimens were transported to Sierra Leone for diagnostic confirmation, which significantly increased the duration of the reporting and turnaround time for case confirmation, especially in a Lassa fever-endemic country, such as Liberia. Limited training of laboratory personnel in molecular diagnostic techniques, and laboratory response in a health emergency, were also a major challenge.

Disease surveillance on the national level has been limited to a paper-based system. Protracted turnaround time and delay in reporting has been a key diagnostic and service delivery challenge in most areas, predominantly in rural settings. Surveillance of diseases has been limited to those in humans and not in animals. In the recent EVD outbreak, active case findings were primarily based on the assumptions of humans as the portals for sporadic outbreaks or re-emerging infections. EVD being a zoonotic disease, its surveillance in animals such as bats, monkeys and other primates should also receive adequate attention from the laboratory diagnostic and public health surveillance systems. Furthermore, hunting communities should also be considered as sources of information gathering and active case findings.

As with our West African neighbours and other countries in sub-Saharan Africa, Liberians have close cultural ties and extended families across the porous borders. Cultural practices, such as eating bush meat (including monkeys, bats, gorillas, etc.), are common cultural norms which could expose communities to zoonotic-associated diseases, such as EVD.^8^ Religious practices such as bathing the dead, wake-keeping for the deceased, gathering in groups and rituals for worship, and secret societies for men and women regarding cultural initiation, also predispose communities by creating favourable portals and suitable enabling environments for the transmission of emerging infectious diseases. For these reasons, the development and availability of diagnostic platforms remain paramount for rapid detection and control of emerging and re-emerging diseases.
